# Use of modern contraceptive methods and pregnancy planning: a cohort study

**DOI:** 10.11606/s1518-8787.2025059006248

**Published:** 2025-04-28

**Authors:** Laísa Rodrigues Moreira, Fernanda Ewerling, Andréa Dâmaso Bertoldi, Mariângela Freitas Silveira

**Affiliations:** I Universidade Federal de Pelotas. Programa de Pós-Graduação em Epidemiologia. Pelotas, RS, Brasil; II Universidade Federal de Pelotas. Centro Internacional de Equidade em Saúde. Pelotas, RS, Brasil

**Keywords:** Contraception, Unplanned Pregnancy, Family Planning, Socioeconomic Factors, Health Inequalities

## Abstract

To investigate the association between unplanned pregnancy and use of modern contraceptive methods at 3-, 12-, and 24-months postpartum using an intersectional approach for examining inequalities, in the 2015 Pelotas birth cohort, Brazil.

We evaluated the use of modern contraceptives after delivery, comparing women who had planned and unplanned pregnancies in 2015. The number of women included in this study was: 4,021, 3,687, and 3,558, at 3-, 12-, and 24-month postpartum follow-ups, respectively. Analyses were restricted to women who needed contraception by the time of each postpartum follow-up interview. Sociodemographic indicators were also investigated. Descriptive (absolute and relative frequencies), bivariate, and multivariate statistical analyses were conducted. These analyses included the main exposure and the sociodemographic variables. In the three follow-up interviews, double stratification was used to investigate for intersectionalities between pregnancy planning and family income, and pregnancy planning and living with partner.

Women who had unplanned pregnancies presented the lowest use of modern contraceptives. A negative association between unplanned pregnancy and use of modern contraception was found at 3- (PR = 0.97, 95%CI: 0.95–0.99) and 24-months postpartum (PR = 0.96, 95%CI: 0.94–0.98). In general, women who did not live with a partner, with lower schooling level, and who were 35 or older presented lower use of modern contraception. Women who had unplanned pregnancies with lower family income and who did not live with a partner presented a lower prevalence of modern contraceptive use.

Despite the possible benefits of the use of modern contraception in the postpartum period, women who had unplanned pregnancies presented the lowest prevalence. No consistent pattern was identified for this association over time. It is necessary to consider important sociodemographic factors such as living with partner as well as possible pathways to reduce inequalities.

## INTRODUCTION

Despite the availability of free contraceptive methods in Brazil, over half of the pregnancies (55.4%) are unintended^11^. Globally, it was estimated that 40% of pregnancies were unintended in 2012, with Latin America and the Caribbean countries presenting the highest rates (56%)^2^. Unplanned pregnancies have significant financial, personal, and interpersonal implications for women and their families. Unplanned pregnancies may also affect the woman’s mental health and have negative impacts on the time and number of contacts with the health system (such as late initiation of antenatal care) and breastfeeding^3–6^.

The use of modern contraceptive methods in postpartum by women whose pregnancy was unplanned is not a well-established subject in the scientific literature, despite potential implications in reproductive and infant health^7–11^. Examples of modern contraceptive methods are oral contraceptives, condoms, injectables, sterilization, and intrauterine devices. Inadequate or inconsistent use of contraception during the postpartum may lead to short pregnancy intervals, unplanned pregnancies, and possible negative maternal and children health outcomes^4 , 5 , 12 , 13^. The World Health Organization recommends women who give birth to wait at least 24 months to have another pregnancy^12^. Short interpregnancy intervals are associated with preterm birth, low birth weight, and subsequent unplanned pregnancies^4 , 5 , 12 , 13^.

In this context, assessing the association between unplanned pregnancy and use of modern contraceptive methods in postpartum is crucial to help healthcare providers, policymakers, and stakeholders implement adequate policies and strategies to promote women’s sexual and reproductive rights in different postpartum periods. Furthermore, the importance of family income and indicators such as living with a partner is well-established in the scientific literature regarding sexual and reproductive health and inequalities in healthcare. However, studies regarding intersectionalities between reproductive health outcomes and such indicators are scarce, despite their potential social and reproductive health implications. Thus, we aim to study the association between unplanned pregnancy and use of modern contraceptive methods at 3-, 12-, and 24-months postpartum, in the 2015 Pelotas birth cohort, Brazil. We also used an intersectional approach to examine inequalities between pregnancy planning and family income, and pregnancy planning and living with partner at 3-, 12-, and 24-months postpartum.

## METHODS

We used data from the 2015 Pelotas birth cohort, gathered at the perinatal study and the 3-, 12-, and 24-month postpartum follow-ups. The birth cohort provides information on women who gave birth to live children in hospitals from Pelotas, Brazil, in 2015. Nearly 98% of all deliveries in Pelotas occur in hospitals^14^. For this study, the analyses were restricted to those who needed contraception by the time of each postpartum follow-up interview (at 3-, 12-, and 24-month postpartum, separately). Women were considered in need of contraception if they were not pregnant and did not want to get pregnant soon. During the perinatal study and at the 3-, 12-, and 24-month postpartum follow-ups, the response rates for the Pelotas birth cohort child participants were 98.7%, 97.2%, 95.4%, and 95,4%, respectively. The parturient were interviewed during the perinatal study (n = 4,219), but at the 3-, 12-, and 24-month postpartum follow-ups other caregivers could answer the questionnaire. However, only data from interviews in which the parturient was the respondent were included in these analyses. Women were asked whether they wanted to get pregnant soon at the 12- and 24-month postpartum follow-ups, but this information was not collected at the 3-month follow-up. After applying the inclusion criteria, the number (with the correspondent percentage from perinatal study) of women included in this study was: 4,021 (95.3%), 3,687 (87.4%), and 3,558 (84,3%) at 3-, 12-, and 24-month postpartum follow-ups, respectively.

The Pelotas birth cohorts have standardized methodology and data collection procedures, warranting the comparison between the follow-ups. Details about the 2015 cohort are available in another study^15^.

### Definitions: Outcomes, Main Exposure, and Other Adjustment Variables

The following question was used as a filter to collect the outcomes data: “Are you doing anything to avoid getting pregnant (contraception)?” If the women answered “Yes,” information about which contraceptive method the women were using was collected. The contraceptives were classified as modern or traditional according to Hubacher and Trussell^16^, which defines modern contraceptives as technological advances designed to enable couples to have sexual intercourse at any mutually-desired time while avoiding pregnancy. Modern methods include: sterilization (male and female); intrauterine devices; subdermal implants; oral contraceptives; condoms (male and female); injectables; emergency contraceptive pills; patches; and vaginal rings. Traditional contraceptive methods include approaches based on sexual abstinence during the fertile period, withdrawal, and lactational amenorrhea^16^. The outcomes of interest were use of modern contraceptive methods at 3-, 12-, and 24-months postpartum, which were operationalized as dichotomous variables (No/Yes).

The main exposure was collected during the perinatal study of the 2015 Pelotas birth cohort by asking the women: “Did you plan on having this child or did you get pregnant by accident?” The possible answers were: “(1) planned,” “(2) by accident,” “(3) more or less” In the analysis of this study, the main exposure was investigated into two categories: “(0) planned”; “(1) by accident/more or less.” Women who were in the category “(1) by accident/more or less” were considered as having unplanned pregnancies.

Sociodemographic indicators associated with the use of modern contraception were also investigated. These indicators were collected in the perinatal study. The sociodemographic indicators were: family income in quintiles (being Q1 the 20% poorest families and Q5 the 20% richest); maternal schooling in years (0–4; 5–8; 9+); maternal employment during pregnancy (No; Yes); maternal age in years (≤ 19; 20–24; 25–29; 30–34; 35+); self-reported maternal skin color (White; Brown/Mixed-race; Black; Yellow; Indigenous); and whether the woman was living with a partner (No; Yes).

### Data Analysis

We evaluated modern contraceptive use at 3-, 12-, and 24 months after delivery in women who needed contraception by the time of each postpartum follow-up interview, comparing those who had planned and unplanned pregnancies. Descriptive (absolute and relative frequencies), bivariate, and multivariate statistical analyses were conducted.

The multivariate statistical analyses included the outcome use of modern contraceptive methods as well as the main exposure, pregnancy planning, and the sociodemographic variables simultaneously. These analyses were independently conducted for each follow-up (at 3-, 12-, and 24 months postpartum). For the bivariate and multivariate statistical analyses, we used Poisson regression with robust variance to estimate the associations^17^, generating the prevalence ratio (PR), a 95% confidence interval (95%CI), and a p-value, obtained by the Wald test. A linear trend test was also used. To evaluate the intersectionality between pregnancy planning and family income, and between pregnancy planning and living with a partner, we performed double stratification. As such, it was possible to identify, for example, the prevalence of modern contraceptive use among women who had planned and unplanned pregnancies in each family income quintile, by follow-up. The statistical analyses were conducted using the statistical software Stata 13.1.

### Ethical Aspects

The 2015 birth cohort project was assessed and approved by the School of Physical Education Ethics Committee from the Federal University of Pelotas. Written informed consent was requested for all 2015 cohort follow-ups, and privacy rights were observed. All procedures of this study were carried out in accordance with the 1964 Declaration of Helsinki and its later amendments.

## RESULTS

In the 2015 cohort, around 52% of the women had unplanned pregnancies (Table 1). At the moment of the interview, 12, 75, and 140 women were pregnant at 3-, 12-, and 24-months postpartum, respectively, and were excluded from this study. They were not asked whether the current pregnancy was planned. Nearly 65% of the participants had nine or more years of schooling, and 56% had worked during pregnancy. Most women were aged 20 to 34 years old (71%). White was the most frequent maternal skin color (71%), and 86% of the women were living with a partner in the perinatal period.

**Table 1.** Overall description of pregnancy planning and use of modern contraceptive methods at 3-, 12-, and 24-months postpartum, 2015 Pelotas Birth Cohort (Pelotas, Brazil).

2015 Cohort	
Follow-up	n
	% (95%CI)
Pregnancy planning	
3 Months postpartum	4,020
Planned	47.9 (46.4–49.5)
Unplanned	52.1 (50.5–53.6)
12 Months postpartum	3,686
Planned	47.6 (46.0–49.2)
Unplanned	52.4 (50.8–54.0)
24 Months postpartum	3,558
Planned	48.5 (46.9–50.2)
Unplanned	51.5 (49.8–53.1)
Use of modern contraceptive methods	
3 Months postpartum	4,021
No	12.1 (11.1–13.1)
Yes	87.9 (86.9–88.9)
12 Months postpartum	3,687
No	10.2 (9.2–11.2)
Yes	89.8 (88.8–90.8)
24 Months postpartum	3,558
No	12.2 (11.2–13.3)
Yes	87.8 (86.7–88.8)

95%CI: 95% confidence interval.

A similar prevalence of modern contraceptive use was found at 3-, 12-, and 24-months postpartum (87.9%, 89.8%, and 87.8%, respectively) (Table 1). Women who had unplanned pregnancies presented the lowest use of modern contraceptive methods in all postpartum follow-ups (Table 2, 3 and 4). In the crude analyses, we found a negative association between unplanned pregnancy and use of modern contraception in all follow-ups (Table 2, 3 and 4). At 3-, 12-, and 24-months postpartum, the prevalence of modern contraceptive use among women who had unplanned pregnancies was 9% (PR = 0.91; 95%CI: 0.89–0.94), 5% (PR = 0.95; 95%CI: 0.93–0.97) and 6% lower (PR = 0.94; 95%CI: 0.92–0.96), respectively, than those who planned their pregnancies (Table 2, 3 and 4). However, after adjustment, only at the 3- (PR = 0.97, 95%CI: 0.95–0.99) and 24-months postpartum periods (PR = 0.96, 95%CI:0.94–0.98) these variables remained statistically significant, with a negative association (Table 2 and 4).

**Table 2.** Crude and adjusted analysis between pregnancy planning and use of modern contraceptive methods at Three Months Postpartum, 2015 Pelotas Birth Cohort (Pelotas, Brazil).

2015 Cohort	Three months postpartum n = 4,021						
	Use of modern contraceptive methods						
		Crude			Adjusted		
Variables	%	PR	95%CI	p	PR	95%CI	p
**Pregnancy Planning**				**< 0.001**			**0.010**
**Planned**	**92.0**	**1.00**			**1.00**		
**Unplanned**	**84.2**	**0.91**	**0.89–0.94**		**0.97**	**0.95–0.99**	
Family income (quintiles)				**< 0.001** ^a^			0.231 ^a^
Q1 (lower income)	83.2	1.00			1.00		
Q2	86.3	1.04	0.99–1.08		1.00	0.96–1.04	
Q3	87.9	1.06	1.01–1.10		0.99	0.95–1.03	
Q4	89.5	1.08	1.03–1.12		1.01	0.97–1.05	
> Q5 (higher income)	92.7	1.11	1.07–1.16		1.03	0.98–1.07	
Maternal schooling (years)				**< 0.001** ^a^			**0.001** ^a^
0–4	80.2	1.00			1.00		
5–8	84.9	1.06	1.00–1.12		1.05	1.00–1.11	
≥ 9	90.2	1.12	1.07–1.19		1.09	1.04–1.16	
Mother worked during pregnancy				0.312			0.097
No	87.3	1.00			1.00		
Yes	88.4	1.01	0.99–1.04		0.98	0.95–1.00	
Maternal age (years)				**0.031**			0.073
> 13–19	84.0	1.00			1.00		
> 20–24	88.4	1.05	1.01–1.10		0.99	0.95–1.03	
> 25–29	89.6	1.07	1.02–1.11		0.97	0.93–1.01	
> 30–34	88.9	1.06	1.02–1.10		0.96	0.92–1.01	
> ≥ 35	87.0	1.04	0.99–1.09		0.94	0.90 – 0.99	
Mother skin color				**0.018**			0.718
White	89.1	1.00			1.00		
Brown	85.2	0.96	0.92–0.99		0.99	0.96–1.03	
Black	84.8	0.95	0.92–0.99		1.01	0.97–1.04	
Yellow	93.3	1.05	0.91–1.20		1.11	0.95–1.30	
Indigenous	90.0	1.01	0.82–1.24		1.05	0.84–1.32	
Living with partner				**< 0.001**			**< 0.001**
No	61.8	1.00			1.00		
Yes	92.2	1.49	1.40–1.59		1.47	1.38–1.57	

PR: prevalence ratio; 95%CI: 95% confidence interval.

Note: results in bold: p < 0.05.

^a^ P value of the X² test for linear trend.

**Table 3.** Crude and adjusted analysis between pregnancy planning and use of modern contraceptive methods at 12 Months Postpartum, 2015 Pelotas Birth Cohort (Pelotas, Brazil).

2015 Cohort	12 Months postpartum n = 3,687						
	Use of modern contraceptive methods						
		Crude			Adjusted		
Variables	%	PR	95%CI	p	PR	95%CI	p
**Pregnancy planning**				**< 0.001**			0.173
**Planned**	**92.2**	**1.00**			**1.00**		
**Unplanned**	**87.7**	**0.95**	**0.93–0.97**		**0.99**	**0.96–1.01**	
Family income (quintiles)				**< 0.001** ^a^			**0.011** ^a^
Q1 (lower income)	84.3	1.00			1.00		
Q2	90.5	1.07	1.03–1.12		1.05	1.01–1.10	
Q3	90.4	1.07	1.03–1.12		1.04	1.00–1.08	
Q4	90.9	1.08	1.04–1.12		1.05	1.00–1.09	
Q5 (higher income)	93.1	1.10	1.06–1.15		1.07	1.03–1.12	
Maternal schooling (years)				**< 0.001** ^a^			**0.021** ^a^
0–4	84.9	1.00			1.00		
5–8	87.8	1.03	0.98–1.09		1.02	0.97–1.07	
≥ 9	91.3	1.08	1.02–1.13		1.05	1.00–1.10	
Mother worked during pregnancy				0.288			0.538
No	89.3	1.00			1.00		
Yes	90.3	1.01	0.99–1.03		0.99	0.97–1.02	
Maternal age (years)				0.054			**0.003**
13–19	89.1	1.00			1.00		
20–24	90.0	1.01	0.97–1.05		0.97	0.93–1.01	
25–29	92.0	1.03	1.00–1.07		0.97	0.93–1.01	
30–34	89.6	1.01	0.97–1.04		0.94	0.90–0.98	
≥ 35	87.2	0.98	0.94–1.02		0.92	0.88–0.96	
Mother skin color				**< 0.001**			**< 0.001**
White	90.0	1.00			1.00		
Brown	88.5	0.98	0.95–1.02		1.01	0.97–1.05	
Black	90.0	1.00	0.97–1.03		1.04	1.01–1.07	
Yellow	100.0	1.11	1.10–1.13		1.16	1.10–1.21	
Indigenous	88.9	0.99	0.78–1.24		1.03	0.82–1.30	
Living with partner				**< 0.001**			**< 0.001**
No	73.4	1.00			1.00		
Yes	92.6	1.26	1.20–1.33		1.26	1.19–1.33	

PR: prevalence ratio; 95%CI: 95% confidence interval.

Note: results in bold: p < 0.05.

^a^ P value of the X² test for linear trend.

**Table 4.** Crude and adjusted analysis between pregnancy planning and use of modern contraceptive methods at 24 Months Postpartum, 2015 Pelotas Birth Cohort (Pelotas, Brazil).

2015 Cohort	24 Months postpartum n = 3,558						
	Use of modern contraceptive methods						
Variables		Crude			Adjusted		
	%	PR	95%CI	p	PR	95%CI	p
**Pregnancy planning**				**< 0.001**			**0.001**
**Planned**	**90.7**	**1.00**			**1.00**		
**Unplanned**	**85.0**	**0.94**	**0.92–0.96**		**0.96**	**0.94–0.98**	
Family income (quintiles)				**0.009** ^a^			0.874 ^a^
Q1 (lower income)	84.8	1.00			1.00		
Q2	88.0	1.04	1.00–1.08		1.02	0.98–1.06	
Q3	88.3	1.04	1.00–1.09		1.00	0.96–1.05	
Q4	87.5	1.03	0.99–1.08		0.99	0.94–1.04	
Q5 (higher income)	90.3	1.06	1.02–1.11		1.01	0.96–1.06	
Maternal schooling (years)				**< 0.001** ^a^			0.058 ^a^
0–4	82.9	1.00			1.00		
5–8	85.9	1.04	0.98–1.10		1.03	0.97–1.09	
≥ 9	89.1	1.07	1.02–1.13		1.05	0.99–1.11	
Mother worked during pregnancy				**0.028**			0.183
No	86.4	1.00			1.00		
Yes	88.9	1.03	1.00–1.05		1.02	0.99–1.05	
Maternal age (years)				0.184			0.060
13–19	86.9	1.00			1.00		
20–24	87.6	1.01	0.97–1.05		0.97	0.93–1.02	
25–29	89.6	1.03	0.99–1.07		0.97	0.93–1.02	
30–34	88.4	1.02	0.97–1.06		0.96	0.91–1.01	
≥ 35	85.2	0.98	0.93–1.03		0.93	0.88–0.98	
Mother skin color				0.168			0.746
White	88.6	1.00			1.00		
Brown	85.5	0.96	0.93–1.00		0.98	0.94–1.02	
Black	86.1	0.97	0.94–1.01		1.00	0.96–1.04	
Yellow	83.3	0.94	0.73–1.21		0.97	0.76–1.24	
Indigenous	66.7	0.75	0.47–1.19		0.78	0.49–1.22	
Living with partner				**< 0.001**			**< 0.001**
No	76.8	1.00			1.00		
Yes	89.5	1.17	1.11–1.23		1.15	1.09–1.21	

PR: prevalence ratio; 95%CI: 95% confidence interval.

Note: results in bold: p < 0.05.

^a^ P value of the X² test for linear trend.

**Figure 1.**
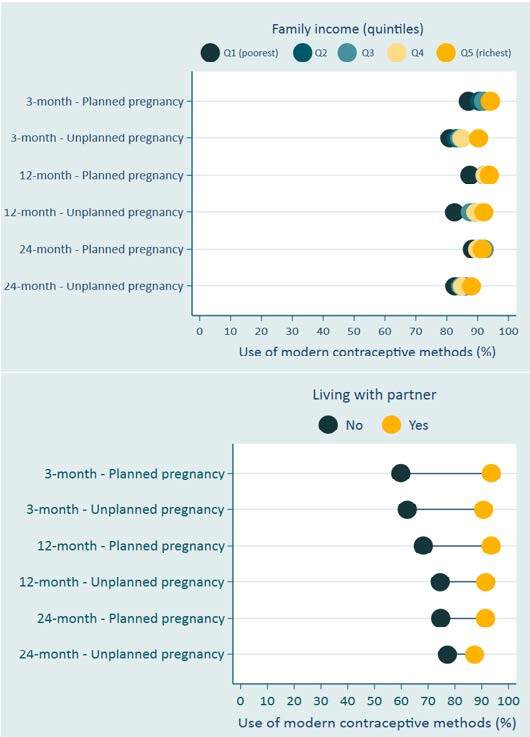
Prevalence of use of modern contraceptive methods according to living with partner, family income (in quintiles), and pregnancy planning at 3-, 12-, and 24-month postpartum, 2015 Pelotas birth cohort (Pelotas, Brazil).

Women who did not live with partners presented lower prevalence of modern contraceptive use in all follow-ups (Table 2, 3 and 4). At three months postpartum, women with lower schooling levels also presented lower use of modern contraception (Table 2). At 12 months follow-up, women that were not living with their partner, with lower income and schooling, aged 35 years old or more, and with Black skin color were the subgroups with significantly lower prevalence of modern contraceptive use (Table 3). In general, women who had unplanned pregnancies with lower family income presented a lower prevalence of modern contraceptive use (around 82%) (Figure 1). Among women who had planned pregnancies, the highest-income women generally presented the highest prevalence of modern contraceptive use (prevalence around 93%). Similar patterns were observed in all follow-ups. Family income inequalities on the use of modern contraceptive methods were more pronounced at the 3- and 12-month postpartum follow-ups, especially for women who had unplanned pregnancies. Nevertheless, among the richest women, the lowest prevalence of use of modern contraceptive methods was found for women who had unplanned pregnancies at 24-month postpartum follow-up (88%).

Women who did not live with a partner generally presented lower use of modern contraception at the 3, 12, and 24 months postpartum, regardless of pregnancy planning (Figure 1). However, inequalities were more pronounced, especially at the three months postpartum follow-up, during which the prevalence of modern contraceptive use in women who had planned pregnancy and who did not live with a partner was 59.8%, whereas in women who had planned pregnancy and lived with a partner was 93.6%, for example. In general, among women who did not live with a partner, those who had unplanned pregnancies presented a slightly higher prevalence of modern contraceptive use than those who had planned pregnancies.

## DISCUSSION

We found that women whose pregnancy was unplanned presented a lower prevalence of modern contraceptive use, with this association being significant at the 3- and 24-month postpartum periods. Living with a partner, higher maternal schooling, and age were associated with higher use of modern contraceptive methods. On the other hand, those who had unplanned pregnancies with lower family income and who did not live with a partner presented a lower prevalence of modern contraceptive use.

Sociodemographic differences in the use of modern contraception, especially related to family income and maternal schooling, were noted. The data suggest a reduction in these inequalities over time. The prevalence of modern contraceptive use increased among women with lower family income and schooling levels, while it decreased among those with higher family income and schooling levels.

The best moment to start contraceptive use after childbirth remains undefined^18^. The use of long-acting reversible contraception (LARC, i.e., intrauterine devices or implants) soon after the delivery is often identified as a good option considering the possible benefits for women and their families^19^. Data from 23 Latin American and Caribbean countries evidenced a low prevalence of LARC (below 10% in most countries) and large inequalities in modern contraceptive use, indicating that in some contexts low acceptability of this type of contraceptive methods may be present, as well as barriers to access them^20^.

In this sense, access to a wide range of modern contraceptive methods and information on the suitability of such methods for each woman is important to address the issue of family planning. Our research indicated that around 10% of the women did not use contraception; as such, identifying the reasons for this is fundamental to overcome possible barriers. In a study across 47 low and middle-income countries, the most frequently reported reasons for not using contraception included health concerns, infrequent sex, and opposition to contraception. Potential inequalities within these countries, such as wealth-related inequalities, might also play a role^21^.

Maternal health-related inequalities were observed in the Pelotas birth cohorts (1982, 1993, 2004, and 2015) with Black or Brown-skinned mothers more likely to be 19 years old or younger at the time of childbirth, for example^22^. The use of modern contraceptive methods in the postpartum period prevents outcomes such as short interpregnancy intervals which is related to low birthweight and preterm birth^12 , 13^. In the 2015 Pelotas birth cohort, the prevalences of low birthweight and preterm birth were 8.3% and 13.8%, respectively, and were also associated with sociodemographic factors such as family income and maternal skin color^23^.

In this context, other factors such as living with partner are also important. The double stratification results including the indicator “living with partner” suggest that women may believe they have a lower risk of getting pregnant when they do not live with a partner, especially in the first months after delivery, which leads them to not use contraception. Furthermore, among women who do not live with a partner, those who had unplanned pregnancies presented a prevalence of modern contraceptive use slightly higher than those who had planned pregnancy, suggesting that women who do not live with a partner have a lower “risk perception” of becoming pregnant. Infrequent sex is also a commonly cited reason for the nonuse of contraceptive methods.

Maternal age was another relevant factor. Recurrent unplanned pregnancies, for example, are frequently studied in teenagers, although these pregnancies also occur in other age groups^7 , 24 , 25^. In this study, the lowest prevalence of modern contraceptive use was found among women aged 35 years or more. Moreover, a study with data from the 1982, 1993, 2004, and 2015 Pelotas birth cohorts, in their sociodemographic description, have demonstrated an increase in mothers with 35 or more years old, with inequalities related to maternal skin color (the increase over time occurred in white women)^22^. It is also possible that some women in this age group have comorbidities that complicate their choices regarding modern contraceptive methods, making health concerns an important reason for nonuse. Identifying the most vulnerable subgroups and guaranteeing access to contraception to postpartum women in need of family planning are fundamental steps to prevent unplanned pregnancies.

One of the strengths of this study is its temporality, as the exposure was collected in the perinatal study and the outcomes were assessed over time (at 3-, 12-, and 24-months postpartum) using data from a birth cohort. Furthermore, the 2015 Pelotas birth cohort uses standardized procedures at all steps. However, the lack of information on abortions is one of our limitations, which may lead to underestimations in the prevalence of unplanned pregnancies. Furthermore, given the differences in the definitions and measures of unplanned pregnancies in the scientific literature, it is difficult to compare our results with the findings from other studies^26 , 27^. However, the results found in Pelotas are probably comparable to other settings with similar sociodemographic characteristics. Another limitation is that at the 3-month postpartum interview, the participants were not asked whether they wanted to get pregnant soon. At three months postpartum, women who were exclusively breastfeeding would be protected by the lactational amenorrhoea method (LAM), which is a traditional contraceptive method according to the definitions of this study^16^. This method protects 98% of pregnancies among women who are exclusively breastfeeding, amenorrhoeic, and less than six months postpartum^28^. In Pelotas, nearly 45% of the 2015 birth cohort participants reported exclusive breastfeeding at three months^29^. As such, the effectiveness of LAM would be relatively high for these women.

Despite their benefits, many women who need contraception in the postpartum period do not use modern methods^18^. Our study revealed that, in Pelotas, modern contraceptive use during the postpartum period was lower among women who had an unplanned pregnancy, which may indicate a perpetuation of non-preventive behavior. However, we did not find a consistent pattern for this association over time, as it was true only at the 3- and 24-months postpartum periods, but not at the 12-month follow-up. Although the prevalence ratios suggest a slight effect, their importance for public health is evident. All women in need of contraception should have access to contraceptive methods and have their sexual and reproductive rights guaranteed not only after delivery but also throughout their lives. In this sense, it is crucial to consider the complexity of different postpartum periods as well as to investigate this association in different settings. Furthermore, it is essential for basic healthcare services to address women’s needs beyond infant care, such as maternal depression, which is common in the postpartum period^30^. These interactions with the healthcare system provide critical opportunities to offer comprehensive and universal health care, strengthen the family healthcare network, and support the reproductive needs and rights of mothers.
